# The association between physical exercise and the subjective well-being of older adults—the mediating role of three-dimensional capital

**DOI:** 10.3389/fpsyg.2025.1703314

**Published:** 2026-01-12

**Authors:** Yongchao Lang

**Affiliations:** School of Economics and Management, Wuhan Sports University, Wuhan, Hubei, China

**Keywords:** health capital, older adults, physical exercise, psychological capital, social capital, subjective well-being

## Abstract

**Introduction:**

As the issue of global population aging intensifies, the enhancement of subjective well-being (SWB) among older adults has become a central focus of societal discourse. This concern is particularly pressing in China, where the older population is not only large but also growing at a rapid pace, thereby placing increased demands on the social security system and public health policies. Although existing studies predominantly explore the associations between external factors such as economic security, healthcare resources, and social support on older adults’ SWB, the mechanisms through which physical exercise, as an active health management strategy, is associated with SWB remain insufficiently explored.

**Methods:**

Drawing on capital theory and positive psychology, this study integrates health capital, social capital, and psychological capital to construct a “three-dimensional capital” analytical framework. Using data from the China Family Panel Studies (CFPS), we systematically examine the associations between physical exercise participation and older adults’ SWB and assess the parallel mediating paths of the three-dimensional capital.

**Results:**

The findings reveal that participation in physical exercise is significantly associated with higher overall SWB, with higher levels in the specific SWB indicators of happiness and life satisfaction, alongside a lower depression index. In terms of the underlying mechanisms, physical exercise shows statistically significant indirect associations through three pathways—health capital, social capital, and psychological capital. Heterogeneity analysis further reveals that the associations between physical exercise and SWB differ across groups defined by gender, urban–rural residence, and marital status.

**Discussion:**

Overall, the study suggests that physical exercise is associated with the SWB of older adults through the joint roles of health, social, and psychological capitals, thereby extending the explanation of these associations from a single-capital pathway to a more comprehensive theoretical perspective grounded in three-dimensional capital. Accordingly, it is recommended that policies related to community support, health management, and psychological interventions be implemented to support pathways for older adults’ participation in physical exercise that are linked to improved SWB in an aging society through the synergistic accumulation of multidimensional capitals.

## 1 Introduction

Since the turn of the 21st century, the global aging trend has accelerated, becoming a significant challenge for both socio-economic development and public policy (Shaik et al., 2024). According to the latest statistics, by the end of 2024, China’s population aged 60 and above had reached approximately 310 million, accounting for 22.0% of the total national population. Among them, the population aged 65 and above numbered about 220 million, representing 15.6%. At the same time, the United Nations predicts that the global older population will continue to grow rapidly in the coming decades, further highlighting the universality and severity of the aging issue. As such, how to effectively improve the well-being of older adults and genuinely enhance their quality of life has become an urgent issue, not only in China but globally (Marzo et al., 2023).

Well-being can be considered from both objective and subjective dimensions. Objective welfare primarily involves economic security, healthcare services, and social support, while SWB pertains to psychological health, life satisfaction, and personal happiness (Diener and Seligman, 2004). As socio-economic development and changes in family structures evolve, the importance of SWB within the overall well-being of older adults has become increasingly prominent (Huang et al., 2022). However, compared to objective welfare, improvements in SWB are often overlooked. Enhancing SWB not only contributes to the physical and mental health of older adults but also has profound implications for the sustainable development of society and the economy (Owen et al., 2022). Existing research predominantly focuses on the associations between external factors such as public service policies (Diener et al., 2018), intergenerational family relations (Katz, 2009), family wealth (Jantsch et al., 2024), urban development (Ding et al., 2024), and family companionship (Cheng and Yan, 2021) and older adults’ SWB. However, physical exercise, as a proactive approach to health promotion, may play a unique role in older adults’ SWB. Despite the extensive literature examining the associations between physical exercise and outcomes among older adults, including improvements in physical health, alleviation of depression, reduction of mental stress, and enhancement of cognitive functions (Izquierdo and Singh, 2023; Wong et al., 2023), research on the relationship between exercise participation and older adults’ SWB remains inconclusive. More importantly, most existing studies tend to focus on a single pathway (e.g., physical health improvement or social interaction) and lack an integrated theoretical framework that simultaneously encompasses physical, social, and psychological dimensions to systematically explicate the underlying mechanisms (Lera-López et al., 2017). Therefore, exploring how physical exercise is associated with the SWB of older adults is not only of significant academic value but also of practical importance in addressing the challenges posed by an aging population.

To this end, this study utilizes large-scale social survey data with broad representativeness—the China Family Panel Studies (CFPS)—to measure the SWB of older adults using three SWB indicators: happiness, life satisfaction, and the depression index. By constructing a multivariate linear regression model, this study systematically analyzes the associations between physical exercise participation and SWB. Furthermore, to overcome the limitations of single-pathway explanations, this study constructs a “three-dimensional capital” mediation framework that incorporates health capital, social capital, and psychological capital. This framework is designed to move beyond traditional single-capital perspectives by uncovering potential synergistic and parallel pathways among the three forms of capital in the relationship between physical exercise and SWB, thereby offering a more comprehensive and systematic mechanism-based explanation. This study aims to deepen the understanding of the mechanisms influencing the SWB of older adults, providing empirical evidence for the formulation of targeted policies, the optimization of elderly care services, and the enhancement of the older adults’ quality of life, while offering important insights for promoting social harmony and sustainable development.

## 2 Literature review and research hypotheses

### 2.1 Older adults’ SWB and measurement methods

SWB refers to an individual’s internal evaluation of life satisfaction or fulfillment, which, in contrast to objective material welfare, more accurately reflects an individual’s actual well-being (Diener and Suh, 1997). As a vital supplement to the traditional economic welfare system, SWB has increasingly attracted the attention of both governments and scholars. Enhancing the SWB of the population has thus become one of the central goals of societal development.

Compared to younger groups, older adults are considered a relatively vulnerable demographic due to characteristics such as advanced age, declining health, and restricted mobility. Consequently, when examining the factors that are associated with the SWB of older adults, scholars focus on critical dimensions such as health status, community environment, intergenerational support, economic security, and social welfare (Chyi and Mao, 2012; Huang et al., 2022; Moreno et al., 2014). Regarding health status, as individuals age, their physiological functions gradually decline, increasing the risk of severe diseases and disability. The deterioration of physical health and functional ability often accompanies a decline in psychological and social adaptability, which can negatively be associated with the SWB of older adults (Steptoe et al., 2015). On the family and social level, multiple role transitions, such as the shift from employment to retirement, can bring economic pressures, and a change in familial authority or the loss of loved ones can lead to a “role loss effect,” further affecting the SWB of older adults (Lin et al., 2023; Yuan et al., 2024). From the perspective of the community environment, Shen (2014) found that the quantity of community organizations and facilities has a positive impact on the mental health of middle-aged and elderly individuals.

SWB reflects an individual’s comprehensive experiences and evaluations across various life domains. In exploring methods to quantify SWB, Fitouss et al. (2011) distinguished between long-term cognitive evaluations of life quality and immediate emotional experiences. They proposed measuring SWB using conceptual components: life satisfaction, positive emotions, and negative emotions (Fitouss et al., 2011). Seligman (2011) developed a multi-component model that includes positive emotions, engagement, relationships, meaning, and achievement. Currently, there is a consensus within the academic community that the measurement of SWB should reflect lower levels of negative emotions and higher levels of positive experiences, emphasizing the distinction between positive and negative feelings (Schlossarek et al., 2024). Specifically, depression is commonly used as an indicator for assessing negative emotions, while happiness and life satisfaction are widely used indicators of positive experiences. These indicators comprehensively reflect an individual’s life state, offering strong representativeness, comparability, and scientific validity. Therefore, this study selects the depression index, happiness, and life satisfaction as three SWB indicators among older adults.

### 2.2 Research hypotheses on physical exercise and older adults’ SWB

Physical exercise refers to physical activities designed to improve bodily fitness, characterized by planned, repetitive, and purposeful movements. Existing research has documented positive associations between physical exercise and common SWB indicators, such as happiness and life satisfaction, with potential links operating through an individual’s physiological functions, psychological state, and emotional experiences (Fang et al., 2024; Pedersen and Saltin, 2015). In contrast, a lack of regular physical activity may be associated with more frequent negative emotional experiences. Studies have shown that individuals who engage in regular physical exercise report higher levels of life satisfaction compared to those who do not exercise (Stubbe et al., 2007), with higher reported life satisfaction among exercisers (Bae, 2022). In the context of negative emotions, research has highlighted that regular physical exercise is associated with fewer negative emotional experiences, often resulting in reduced levels of anxiety and depression before and after exercise (Martín-Rodríguez et al., 2024).

Studies focused on older adults indicate that physical exercise is associated with lower feelings of loneliness, fewer negative emotions, and higher life satisfaction and happiness, thereby being linked to both physical and mental health and supporting the realization of positive ageing (Sebastiao and Mirda, 2021; Szychowska and Drygas, 2022). Despite using different indicators to reflect SWB among older adults, research findings consistently point to the significant positive impact of physical exercise. For example, Lu et al. (2022) found that, compared to sedentary older adults, those who regularly engage in physical exercise exhibit a significant reduction in cognitive decline. Participation in physical activities stimulates the secretion of neurotransmitters like endorphins, improving mood, alleviating anxiety and depressive symptoms, and higher self-efficacy and life satisfaction among older adults. Mammen and Faulkner (2013), through a literature review, found that physical activity can effectively prevent the onset of depression in older adults. Similarly, Holmquist et al. (2017) identified a decline in physical activity capacity as a major factor contributing to depression in older adults aged 70 and above.

In summary, a thorough analysis of existing research clearly reveals a significant positive association between physical exercise participation and the SWB of older adults. Whether in terms of increased happiness and life satisfaction or effective alleviation of negative emotions (such as depression), physical exercise demonstrates a consistent positive relationship with these SWB indicators. Based on this, the following research hypothesis is proposed:

*H1*: Participation in physical exercise is significantly associated with the SWB of older adults.

### 2.3 Mechanisms by which physical exercise is associated with the SWB of older adults

#### 2.3.1 Health capital

Health capital refers to the cumulative accumulation of physical, mental, and socio-economic resources within an individual or group, encompassing multiple aspects such as the accessibility of healthcare services, economic income, and social support (Schneider-Kamp, 2021). The human capital model of health views health as a form of “durable” capital (Grossman, 2000), meaning that it is a form of human capital formed and enhanced through the maintenance and investment in an individual’s physical and mental state, serving as the material foundation for the formation and appreciation of other forms of human capital (Albert, 2021).

As a crucial means of health management for older adults, physical exercise plays a significant role in disease prevention, delaying aging, and enhancing physical fitness (Pedersen and Saltin, 2015). Wong et al. (2023) noted the impact of physical activity on the physical health and mental health of older adults, emphasizing the positive effects of different types of physical activity in improving functional health, mental health, and cognitive function in older adults. Studies show that moderate physical exercise positively impacts both subjective and objective health in older adults (Buecker et al., 2021). In terms of subjective health, long-term regular moderate-intensity exercise can enhance happiness (Chen et al., 2022), improve mood states (Klimova and Dostalova, 2020), and improve sleep quality (Fank et al., 2022). In terms of objective health, physical exercise has been linked to stronger myocardial function and better respiratory and neurological function among older adults, and has also been associated with a lower risk of osteoporosis and functional degeneration (Soares-Miranda et al., 2014).

The enhancement of health capital further is associated with the SWB of older adults through factors such as economic income, social participation, and subjective attitudes (Steptoe et al., 2015). Based on the health demand model, health is regarded as the core of human capital, possessing both consumption and investment characteristics. This model suggests that physical and mental health is the foundation for participation in socio-economic activities and the realization of self-worth, and is associated with the time and energy devoted to market labor (e.g., employment) and non-market activities (e.g., socializing, volunteering), thereby determining the ability to create social and economic value (Downward et al., 2020). For older adults, health is not only the cornerstone of quality of life in later years but also a crucial driver for their continued contribution to society and the realization of self-worth, significantly promoting the enhancement of their SWB (Huang et al., 2022). The role of health capital in supporting favorable SWB outcomes is also reflected in more favorable values of the SWB indicators (e.g., higher happiness and life satisfaction, and a lower depression index). For example, An et al. (2020) found that older adults with better health status reported higher levels of happiness and life satisfaction. In contrast, Missler et al. (2011) discovered in a study on older adults’ death anxiety that physical health status was significantly negatively correlated with death anxiety.

Based on the above analysis, the positive impact of physical exercise on the SWB of older adults is, to some extent, dependent on the mediating role of health capital. To further verify this relationship, the following research hypothesis is proposed:

*H2*: Health capital mediates the relationship between physical exercise and SWB in older adults.

#### 2.3.2 Social capital

Social capital refers to resources accumulated through social networks, trust, and shared norms. It extends beyond the accumulation of resources at the individual or group level and is a core characteristic of social organizations (Kiechel, 2000).

As an active social activity, physical exercise is viewed by exercise psychology as a key means that may be associated with social support and participation. Participation in physical exercise not only helps individuals establish positive interpersonal networks but also enables them to gain extensive social support through interaction, thus promoting the accumulation of social capital (Perks, 2007). Furthermore, existing research emphasizes that the frequency of physical exercise participation is positively correlated with the development of an individual’s social interaction skills. High-frequency physical interactions not only optimize one’s popularity in social circles but also significantly increase the total amount of social capital an individual can access (Di Bartolomeo and Papa, 2019). Moreover, recent studies have started to analyze the social effects of physical exercise participation among older adults, leading to valuable findings. Research has shown that physical exercise not only improves the health of elderly individuals but also expands their social networks, thereby enhancing their social participation from multiple aspects—ability, willingness, and opportunity (Szychowska and Drygas, 2022). Physical exercise and recreational activities serve as important social interaction mechanisms for older adults (Hornby-Turner et al., 2017). Rebuilding stable and positive social relationships in later life through sports-based socializing may be important for creating a supportive environment necessary for healthy aging.

Studies indicate that the accumulation of social capital not only promotes positive factors such as self-rated health, class identity, economic income, and happiness but also significantly reduces negative emotions such as depression through the social attributes of physical activities (Arachchi and Managi, 2023; Teychenne et al., 2008). For older adults, with the cessation of employment and the shrinking of their social circles, they increasingly face difficult life circumstances (Fang and Yi, 2025). However, a strong social network may be linked to maintaining a higher level of happiness among older adults (Yang et al., 2024). According to the stress-buffering model (Cacioppo and Hawkey, 2009), rich social capital can significantly buffer the impact of negative psychological emotions when older adults face stressful situations or anticipated stressful events. A robust social network provides emotional comfort and practical assistance (Sun and Lu, 2020), enabling older adults to maintain a positive attitude when confronted with major life events (Cattell, 2001).

In conclusion, physical exercise is associated with social capital through the establishment and expansion of social connections, thereby improving the elderly’s self-rated health, happiness, and life satisfaction, as reflected in the three SWB indicators. Based on this, the following research hypothesis is proposed:

*H3*: Social capital mediates the relationship between physical exercise and SWB in older adults.

#### 2.3.3 Psychological capital

Psychological capital refers to a positive psychological state that individuals exhibit during their growth and development, encompassing subjective attitudes and cognitions such as self-confidence, hope, optimism, resilience, and emotional regulation. It is a form of positive psychological resource that surpasses social capital and human capital (Luthans et al., 2010). Building on this, subsequent studies have further emphasized that psychological capital is characterized by salient positive self-evaluation and future orientation, with its core lying in individuals’ confidence in their own capabilities, their faith in future life, and their ability to maintain a positive stance when facing adversity (Luthans and Youssef-Morgan, 2017). In empirical research based on large-scale survey data, scholars have therefore commonly operationalized this latent psychological resource using indicators such as self-efficacy or confidence in the future (Zhao et al., 2022).

Research indicates that individuals who engage in physical exercise tend to exhibit superior psychological attributes such as self-confidence, resilience, hope, and optimism compared to those who do not exercise (Tu, 2020). From a psychological perspective, physical exercise improves individual psychological factors, such as cognitive abilities, self-efficacy, and attention, which in turn help alleviate depressive symptoms (Miller et al., 2019). From a sociological viewpoint, exercise participants develop a greater sense of control and self-regulation, and the communication, interaction, and cooperation inherent in physical activity promote positive emotions, thereby helping to regulate negative emotions (Evans et al., 2017). From the perspective of exercise’s emotional effects, individuals can improve their self-efficacy by receiving positive feedback from enhanced body flexibility and physical abilities following exercise (Jeon and Kim, 2020). In short, physical exercise positively influences an individual’s psychological capital by enhancing positive emotional experiences and emotional effects, alleviating negative emotions such as depression, tension, and anxiety, and thereby fostering a positive psychological state.

For older adults, psychological health issues are particularly prominent due to the decline in physical functioning, cognitive deterioration, and changes in social roles, such as retirement or children leaving home. Physical exercise, as a proactive health-promoting approach, has been associated with better psychological health among older adults and fewer depressive symptoms (Mumba et al., 2021). The accumulation of psychological capital is also associated with higher SWB among older adults. Research by Santisi et al. found a significant positive correlation between psychological capital and SWB indicators such as life satisfaction and happiness (Saman and Wirawan, 2021), suggesting that high levels of psychological capital enable individuals to adapt flexibly to life challenges and support a higher quality of life. For older adults, psychological capital is a core component of their physical and mental health framework. When older adults possess higher self-efficacy, control, and optimism, they tend to report a more stable and positive psychological state. Moreover, psychological capital may shape older adults’ beliefs and risk perception. As a positive psychological resource, psychological capital is associated with greater happiness in the face of life’s adversities, and its cumulative effect may strengthen older adults’ resilience to risks, enabling them to approach life’s challenges with an optimistic mindset and proactive actions, thereby being linked to higher SWB.

In conclusion, physical exercise is associated with older adults’ self-confidence, resilience, optimism, and self-efficacy—key elements of psychological capital—which is linked to more favorable values of the three SWB indicators. Based on this, the following research hypothesis is proposed:

*H4*: Psychological capital mediates the relationship between physical exercise and SWB in older adults.

## 3 Research design

### 3.1 Data sources and processing

This study utilizes data from the China Family Panel Studies (CFPS) conducted by the China Social Science Survey Center at Peking University, specifically from the years 2018, 2020, and 2022. The CFPS collects multi-level socio-economic development data across personal, household, and community dimensions from various regions in China. The sample covers all provinces (autonomous regions and municipalities) in the country, offering a comprehensive and representative view of the changes across multiple sectors of Chinese society, ensuring both representativeness and authority. Given that the target group for this study is older adults, individuals under the age of 60 were excluded from the analysis, as well as cases with missing responses or variables. After these exclusions, the final sample size available for analysis was 15,433 respondents.

### 3.2 Variable setting

#### 3.2.1 Dependent variable: SWB

This study measures subjective well-being by reducing negative emotional experiences and enhancing positive emotional experiences, drawing on existing research methodologies while balancing both negative and positive perceptions among physical exercise participants (Huamei and Wenxin, 2024). Specifically, the depression index, happiness, and life satisfaction are used as the three SWB indicators for older adults: the depression index captures negative emotional experiences, whereas happiness and life satisfaction capture positive subjective evaluations.

The depression index is evaluated using an 8-item depression tendency scale, including the following items: feeling “down, fatigued by any task, having poor sleep, joyful, lonely, happy with life, sad, and unable to continue with life.” The responses to these items, ranging from “never,” “sometimes,” “often,” to “most of the time,” are assigned scores from 0 to 3. Reverse-scored items are inversely coded, yielding a total depression index score with a maximum value of 24. A higher score indicates a greater likelihood of depression, while a lower score suggests better mental health. Happiness is measured using the item “How happy are you?” with scores ranging from 0 to 10. Life satisfaction is measured with the item “How satisfied are you with your life?” where responses ranging from “very dissatisfied” to “very satisfied” are assigned values from 1 to 5.

#### 3.2.2 Core explanatory variable: physical exercise

The frequency of physical exercise is selected as the key indicator to reflect exercise participation. Specifically, the survey includes the question, “How frequently have you participated in physical fitness and recreational activities over the past 12 months?” Based on the survey responses, a binary variable for “exercise participation” is created: if the respondent engages in physical exercise more than zero times, the value is set to 1, indicating participation in physical exercise; otherwise, the value is set to 0, indicating no participation in physical exercise.

#### 3.2.3 Mediating variables

In terms of health capital, self-reported health is used as an indicator, with the survey question: “How would you rate your health status?” The responses, ranging from “unhealthy” to “very healthy,” are assigned scores from 1 to 5. For social capital, an individual’s social relationships are used as a proxy. The survey question is: “How good are your social relationships?” The responses are scored from 0 to 10, with higher scores indicating better social relationships, which correspond to a higher level of social capital. Regarding psychological capital, based on its defining features of positive self-evaluation and future orientation, and taking into account the availability of CFPS data, we use individuals’ confidence in the future as an operational indicator of its core characteristics. Specifically, psychological capital is measured by the survey item “How confident are you about your future?” Responses are scored from 1 to 5, with higher scores indicating greater psychological capital.

#### 3.2.4 Control variables

To more accurately estimate the net association of physical exercise on the SWB of older adults and to control for confounding bias arising from observable characteristics, this study, drawing on previous research, includes a set of control variables closely related to health behaviors and SWB among the elderly. These variables cover core demographic characteristics (Gender, Age), socio-economic status (Educational Level, Relative Income, Household Registration Location), and socio-cultural and family context (Marital Status, Religion), to mitigate potential confounding effects attributable to these factors. The detailed construction of these variables is presented in Table 1.

**Table 1:** Variable design and description.

Variable type	Variable name	Definitions
Dependent variables	Happiness	Happiness feeling from low to high is 0 to 10
	Life satisfaction	From unsatisfactory to very satisfactory, 1 to 5, respectively.
	Depression index	Depression level from low to high is 0 to 24
Independent variables	Physical exercise	Participation = 1, non-participation = 0
Control variables	Gender	Male = 1, Female = 0
	Age	Age selected from the 60 + group
	educational level	Primary schools and below = 1, middle school = 2, high school = 3, college and above = 4
	Marital status	Married = 1, Single = 0
	Religion	Religious = 1, no religion = 0
	Household registration location	Rural = 1, non-rural = 0
	Relative Income	Position of self-assessed income in the local area: very low = 1 ~ very high = 5
Mediating variables	Health capital	Unhealthy = 1 ~ Very healthy = 5
	Social capital	Assign values from 0 to 10 from the lowest to the highest.
	Psychological capital	Assign values from 1 to 5 from very low confidence to very high confidence.

### 3.3 Econometric model construction

In terms of econometric methodology, this study pools the survey samples from three waves (2018, 2020, and 2022) into a cross-sectional dataset. Although the SWB and capital variables involved are ordinally categorized, they contain a sufficient number of categories. Following common practice in social survey research, it is methodologically reasonable to treat them as continuous variables and estimate using Ordinary Least Squares (OLS). The model controls for year-fixed effects, individual and household characteristics, and employs individual-clustered robust standard errors. The basic form of the model is specified as follows:


(1) $${\mathrm{SWB}}_{\mathrm{it}}=\alpha +\beta_{1}{\mathrm{Sport}}_{\mathrm{it}}+\mu^{\prime }C_{\mathrm{it}}+\theta^{\prime }{\mathrm{Year}}_{t}+\delta_{\mathrm{it}}$$

Equation 1 presents the baseline model for examining the association between participation in physical exercise and older adults’ SWB. $SWB_{it}$ denotes the SWB level of individual i in year t , measured by the depression index, happiness, or life satisfaction; $Sport_{it}$ indicates whether the individual participates in physical exercise; $C_{it}$ is the vector of control variables; ${\mathrm{Year}}_{t}\;$ represents the year dummy variables; and $\delta_{it}$ is the random error term.

To further examine whether the association between physical exercise participation and the SWB of older adults operates through the mediating roles of health capital, social capital, and psychological capital, this study uses a stepwise regression method to test the significance of the coefficients. The basic form of the mediating effect model is specified as follows:


(2) $$M_{\mathrm{it}}=\alpha_{1}+\beta_{2}{\mathrm{Sport}}_{\mathrm{it}}+{\mu^{\prime }}_{1}C_{\mathrm{it}}+{\theta^{\prime }}_{1}{\mathrm{Year}}_{t}+u_{\mathrm{it}}$$

(3) $${\mathrm{SWB}}_{\mathrm{it}}=\alpha_{2}+\beta_{3}{\mathrm{Sport}}_{\mathrm{it}}+\beta_{4}M_{\mathrm{it}}+\mu_{2}^{\prime }C_{\mathrm{it}}+\theta_{2}^{\prime }{\mathrm{Year}}_{t}+\epsilon_{\mathrm{it}}$$

In the above equations, $M_{it}$ denotes the mediating variables, which include health capital, social capital, and psychological capital. Coefficients *β_2_*,*β_3_*,*β_4_* are parameters to be estimated, while the remaining variables and symbols are consistent with those defined in the baseline model. The procedure for testing the mediation effects proceeds in three steps: Equation 1 serves as the first step, estimating the total association of physical exercise on SWB. Equations 2, 3 constitute the second and third steps, estimating the relationships involving the mediating variables. To further strengthen the robustness of the mediation tests, following the methodological framework of Hayes (2017) and the practical recommendations of Preacher and Hayes (2008), we complemented the stepwise regression approach with bootstrap inference for indirect effects. Specifically, we conducted a nonparametric cluster bootstrap with resampling with replacement at the individual level, using 5,000 replications and setting a random seed to ensure replicability. We report normal-based (Wald-type) 95% bootstrap confidence intervals constructed from bootstrap standard errors, and use these intervals to assess the statistical significance of the indirect effects.

### 3.4 Descriptive statistics and basic facts

Table 2 provides detailed descriptive statistics for each variable. Compared to older adults who do not participate in physical exercise, those who engage in physical exercise report more favorable values in terms of SWB. Specifically, their average happiness score is 7.94, and their average life satisfaction score is 4.28, both of which are higher than the corresponding scores for non-exercising older adults, with average happiness at 7.56 and life satisfaction at 4.22. Additionally, older adults who participants in physical exercise perform better on the depression index, with an average score of 5.00, which is lower than the average depression index score of 6.28 for non-participants.

**Table 2:** Descriptive statistics of each variable.

Variable	Full sample (*n* = 15,433)			No exercise (*n* = 8,463)			Exercise (*n* = 6,970)		
	Avg.	Min	Max	Avg.	Min	Max	Avg.	Min	Max
Happiness	7.73	0	10	7.56	0	10	7.94	0	10
Life satisfaction	4.25	1	5	4.22	1	5	4.28	1	5
Depression index	5.70	0	24	6.28	0	24	5.00	0	24
Physical exercise	0.45	0	1	0.00	0	0	1.00	1	1
Gender	0.52	0	1	0.50	0	1	0.54	0	1
Age	68.10	60	97	68.12	60	95	68.07	60	97
Educational level	1.57	1	4	1.43	1	4	1.74	1	4
Marital status	0.83	0	1	0.83	0	1	0.83	0	1
Religion	0.04	0	1	0.04	0	1	0.04	0	1
Household registration location	0.68	0	1	0.79	0	1	0.54	0	1
Relative income	3.12	1	5	3.10	1	5	3.15	1	5
Health capital	2.61	1	5	2.53	1	5	2.69	1	5
Social capital	7.37	0	10	7.31	0	10	7.46	0	10
Psychological capital	4.14	1	5	4.09	1	5	4.20	1	5

Furthermore, the overall sample of older adults has an average age of 68.10 years, with a slight predominance of males over females. The overall educational level is relatively low, and the proportion of individuals with rural household registration is high. Eighty-three percent of the participants were married at the time of the survey. For further details on the descriptive statistics of the variables, please refer to Table 2.

## 4 Results and analysis

### 4.1 Baseline regression analysis

The regression results for the baseline model are presented in Table 3. The regression results for Models (1), (2), and (3) indicate that, after controlling for other variables, older adults who participate in physical exercise have a happiness score that is 0.299 points higher, a life satisfaction score that is 0.079 points higher, and a depression index that is 0.824 points lower compared to those who do not engage in physical exercise, with all coefficients being statistically significant at the 1% level. These results suggest that participation in physical exercise is significantly associated with higher SWB among older adults, consistent with H1.

**Table 3:** Associations between physical exercise participation and SWB indicators.

Variable	(1)	(2)	(3)
	Happiness	Life satisfaction	Depression index
Physical exercise	0.299***	0.079***	−0.824***
	(0.037)	(0.015)	(0.077)
Gender	−0.101**	−0.026	−1.043***
	(0.041)	(0.016)	(0.087)
Age	0.025***	0.009***	−0.021***
	(0.003)	(0.001)	(0.007)
Educational level	0.014	−0.059***	−0.389***
	(0.026)	(0.010)	(0.055)
Marital status	0.369***	0.081***	−1.582***
	(0.058)	(0.022)	(0.128)
Religion	−0.009	0.062*	0.237
	(0.095)	(0.035)	(0.212)
Household registration location	−0.278***	0.018	1.166***
	(0.044)	(0.018)	(0.096)
Relative income	0.407***	0.245***	−0.469***
	(0.017)	(0.007)	(0.034)
Year fixed effect	Yes	Yes	Yes
Constant term	4.515***	2.882***	10.695***
	(0.262)	(0.103)	(0.562)
N	15,433	15,433	15,433
*R* ^2^	0.069	0.117	0.101

*p < 0.1, **p < 0.05, ***p < 0.01; cluster-robust standard errors at the individual level in parentheses.

### 4.2 Mechanisms by which physical exercise is associated with the SWB of older adults

To further explore how physical exercise is associated with the SWB of older adults, the study employs a stepwise regression method to systematically test its mechanisms. Specifically, this study aims to sequentially examine the mediating roles of health capital, social capital, and psychological capital in the relationship between physical exercise and SWB, thereby revealing whether the mechanisms for each of these factors are valid and effective.

#### 4.2.1 Health capital

Table 4 presents the results for testing the mediating effect of health capital. In Model (4), the coefficient for physical exercise is significantly positive, indicating that participation in physical exercise is associated with a higher health status among older adults. Model (5) shows that both physical exercise and health capital have significantly positive coefficients, suggesting that physical exercise is associated with higher health capital, which is, in turn, associated with higher happiness among older adults. Similarly, in Model (6), the coefficients for both physical exercise and health capital are significantly positive, indicating that physical exercise is associated with higher life satisfaction through health capital. In Model (7), the coefficients for both physical exercise and health capital are significantly negative, showing that physical exercise is associated with lower depression levels through health capital. In addition, the bootstrap results based on 5,000 resamples are summarized in Table 5: the indirect effects of physical exercise on happiness, life satisfaction, and depression index through health capital are 0.046, 0.013, and −0.166, respectively, with 95% confidence intervals that do not contain 0, further supporting the significance and robustness of the indirect associations through health capital. Overall, participation in physical exercise is associated with more favorable values of the SWB indicators through the indirect pathway via health capital, consistent with H2.

**Table 4:** Mediation effect of health capital.

Variable	(4)	(5)	(6)	(7)
	Health capital	Happiness	Life satisfaction	Depression index
Physical exercise	0.158***	0.252***	0.067***	−0.657***
	(0.022)	(0.036)	(0.015)	(0.073)
Health capital	–	0.294***	0.082***	−1.054***
	–	(0.015)	(0.006)	(0.031)
Control variables	Yes	Yes	Yes	Yes
year fixed effect	Yes	Yes	Yes	Yes
Constant term	2.451***	3.795***	2.682***	13.277***
	(0.158)	(0.261)	(0.104)	(0.537)
N	15,433	15,433	15,433	15,433
*R* ^2^	0.054	0.096	0.130	0.181

***p < 0.01; cluster-robust standard errors at the individual level are in parentheses.

**Table 5:** Bootstrap indirect effects via three mediators (5,000 resamples).

Mediator	Outcome	Indirect effect	Boot SE	95% Bootstrap CI	
				LL	UL
Health capital	Happiness	0.046***	0.007	0.033	0.060
	Life satisfaction	0.013***	0.002	0.009	0.017
	Depression index	−0.166***	0.024	−0.213	−0.119
Social capital	Happiness	0.071***	0.017	0.037	0.105
	Life satisfaction	0.012***	0.003	0.006	0.018
	Depression index	−0.039***	0.010	−0.059	−0.019
Psychological capital	Happiness	0.092***	0.013	0.066	0.119
	Life satisfaction	0.052***	0.007	0.037	0.066
	Depression index	−0.127***	0.019	−0.164	−0.090

CIs are normal-based 95% bootstrap CIs. ***p < 0.01.

#### 4.2.2 Social capital

Table 6 presents the results for testing the mediating effect of social capital. In Model (8), the coefficient for physical exercise is significantly positive, indicating that participation in physical exercise is associated with higher social capital among older adults. In Model (9), both physical exercise and social capital have significantly positive coefficients, suggesting that physical exercise is associated with higher social capital, which is, in turn, associated with higher happiness among older adults. Similarly, in Model (10), the coefficients for both physical exercise and social capital are significantly positive, further indicating that physical exercise is associated with higher life satisfaction through social capital. In Model (11), the coefficients for both physical exercise and social capital are significantly negative, showing that physical exercise is associated with lower depression levels through social capital. In addition, the bootstrap results based on 5,000 resamples are summarized in Table 5: the indirect effects of physical exercise on happiness, life satisfaction, and the depression index through social capital are 0.071, 0.012, and −0.039, respectively. The 95% confidence intervals for all effects do not include 0, further supporting the significance and robustness of the indirect associations through social capital. Overall, physical exercise is significantly associated with the SWB of older adults through the indirect pathway via social capital, consistent with H3.

**Table 6:** Mediation effect of social capital.

Variable	(8)	(9)	(10)	(11)
	Social capital	Happiness	Life satisfaction	Depression index
Physical exercise	0.145***	0.228***	0.067***	−0.784***
	(0.036)	(0.033)	(0.014)	(0.076)
Social capital	–	0.487***	0.084***	−0.271***
	–	(0.010)	(0.004)	(0.019)
Control variables	Yes	Yes	Yes	Yes
Year fixed effect	Yes	Yes	Yes	Yes
Constant term	5.557***	1.808***	2.414***	12.201***
	(0.256)	(0.230)	(0.103)	(0.571)
N	15,433	15,433	15,433	15,433
*R* ^2^	0.026	0.276	0.154	0.116

***p < 0.01; cluster-robust standard errors at the individual level are in parentheses.

#### 4.2.3 Psychological capital

Table 7 presents the results for testing the mediating effect of psychological capital. In Model (12), the coefficient for physical exercise is significantly positive, indicating that participation in physical exercise is associated with higher psychological capital among older adults. In Model (13), both physical exercise and psychological capital have significantly positive coefficients, suggesting that physical exercise is associated with higher psychological capital, which is, in turn, associated with higher happiness among older adults. Similarly, in Model (14), the coefficients for both physical exercise and psychological capital are significantly positive, indicating that physical exercise is associated with higher life satisfaction through psychological capital. In Model (15), the coefficients for both physical exercise and psychological capital are significantly negative, showing that physical exercise is associated with lower depression levels through psychological capital. In addition, the bootstrap results based on 5,000 resamples are summarized in Table 5: the indirect effects of physical exercise on happiness, life satisfaction, and depression index through psychological capital are 0.092, 0.052, and −0.127, respectively. The 95% confidence intervals for all of these effects do not contain 0, further supporting the significance and robustness of the indirect associations through psychological capital. Overall, physical exercise is significantly associated with the SWB indicators of happiness, life satisfaction, and the depression index through the indirect pathway through psychological capital, consistent with H4.

**Table 7:** Mediation effect of psychological capital.

Variable	(12)	(13)	(14)	(15)
	Psychological capital	Happiness	Life satisfaction	Depression index
Physical exercise	0.114***	0.206***	0.028**	−0.697***
	(0.016)	(0.034)	(0.012)	(0.074)
Psychological capital	–	0.813***	0.454***	−1.116***
	–	(0.021)	(0.009)	(0.042)
Control variables	Yes	Yes	Yes	Yes
Year fixed effect	Yes	Yes	Yes	Yes
Constant term	3.266***	1.859***	1.400***	14.338***
	(0.116)	(0.248)	(0.092)	(0.557)
N	15,433	15,433	15,433	15,433
*R* ^2^	0.132	0.190	0.342	0.154

**p < 0.05, ***p < 0.01; cluster-robust standard errors at the individual level are in parentheses.

### 4.3 Heterogeneity analysis of the associations between physical exercise and SWB among older adults

Table 8 presents the results from subgroup regressions by gender, urban/rural residence, and marital status, which provide a descriptive comparison of the direction and magnitude of the exercise–SWB associations across groups. In terms of happiness, the association between physical exercise and happiness differs in point estimates across subgroups. Specifically, physical exercise is associated with higher happiness with relatively larger coefficient estimates for men, rural residents, and unmarried older adults compared to women, urban residents, and married older adults. For life satisfaction, the association between physical exercise and life satisfaction also shows subgroup differences in point estimates. Compared to men, urban residents, and married older adults, women, rural residents, and unmarried older adults show relatively larger positive coefficient estimates between physical exercise and life satisfaction. Regarding the depression index, the association between physical exercise and depression index also varies in magnitude across subgroups. Specifically, women, urban residents, and unmarried older adults show relatively larger negative coefficient estimates compared to men, rural residents, and married older adults.

**Table 8:** Heterogeneity test by gender, urban/rural, and marital status.

Variable	Gender		Urban and rural		Marital status	
	Male	Female	Urban	Rural	Married	Single
Happiness	0.320*** (0.050)	0.283*** (0.055)	0.239*** (0.058)	0.329*** (0.047)	0.279*** (0.040)	0.398*** (0.097)
Life satisfaction	0.076*** (0.020)	0.084*** (0.021)	0.063** (0.025)	0.085*** (0.018)	0.064*** (0.016)	0.155*** (0.037)
Depression index	−0.805*** (0.103)	−0.864*** (0.114)	−1.003*** (0.127)	−0.755*** (0.095)	−0.759*** (0.082)	−1.152*** (0.204)

*p < 0.1, **p < 0.05, ***p < 0.01; cluster-robust standard errors at the individual level in parentheses.

### 4.4 Robustness check

Earlier, this study examined the associations between physical exercise and the SWB of older adults. To validate the robustness of the findings, two analytical strategies were employed in this section: first, by introducing the Ologit model for parameter estimation; and second, by adding potential omitted variables to the baseline specification.

#### 4.4.1 Model substitution

To further strengthen the robustness of the research conclusions, this study re-estimated the original data using the Ologit model (i.e., ordered logistic regression), resulting in Models 16 to 18. The robustness check results presented in Table 9 show that physical exercise is positively associated with happiness and life satisfaction among older adults at the 1% significance level and is negatively associated with their depression index. This finding is consistent with the earlier analysis and further supports the robustness of the estimated associations between physical exercise and older adults’ SWB under an alternative model specification.

**Table 9:** Robustness test: substitution of research models.

Variable	(16)	(17)	(18)
	Happiness	Life satisfaction	Depression index
Physical exercise	0.208***	0.136***	−0.333***
	(0.032)	(0.035)	(0.032)
Control variables	Yes	Yes	Yes
Year fixed effect	Yes	Yes	Yes
N	15,433	15,433	15,433
Pseudo R^2^	0.020	0.055	0.019

*p < 0.1, **p < 0.05, ***p < 0.01; cluster-robust standard errors at the individual level in parentheses.

#### 4.4.2 Addition of potential explanatory variables

To further address potential estimation bias arising from omitted variables, the robustness checks extend the baseline model by additionally controlling for social trust, chronic diseases, medical insurance, and cognitive ability, and re-estimating the regression equations. As reported in Table 10, after including these potential confounders, the direction and significance of the associations between physical exercise and happiness, life satisfaction, and the depression index remain largely consistent with the baseline results, indicating that the core findings of this study are robust to this expanded set of control variables.

**Table 10:** Robustness test: addition of potential explanatory variables.

Variable	(19)	(20)	(21)
	Happiness	Life satisfaction	Depression index
Physical exercise	0.283***	0.078***	−0.830***
	(0.037)	(0.015)	(0.075)
Control variables	Yes	Yes	Yes
Year fixed effect	Yes	Yes	Yes
Constant term	4.342***	2.929***	11.260***
	(0.267)	(0.107)	(0.559)
N	15,186	15,186	15,186
R^2^	0.083	0.123	0.143

*p < 0.1, **p < 0.05, ***p < 0.01; cluster-robust standard errors at the individual level in parentheses.

## 5 Discussion and recommendations

### 5.1 Discussion

#### 5.1.1 The association between physical exercise participation and SWB among older adults

The empirical analysis of this study indicates that participation in physical exercise is significantly associated with higher SWB among older adults in China. This association is reflected not only in higher happiness and life satisfaction but also in lower levels of depressive symptoms. This finding aligns closely with existing conclusions in the field of aging research regarding the psychological effects of physical exercise, further supporting its broad relevance (Zhang Y. S. et al., 2024).

However, this study goes beyond the traditional scope by systematically integrating the three SWB indicators to reveal overall associations between physical exercise and older adults’ SWB within a unified analytical framework. Specifically, physical exercise is associated with both lower negative emotions (such as alleviation of depressive symptoms) and higher psychological resources (such as increases in happiness and life satisfaction), demonstrating a dual effect of “burden reduction” and “gain.” Previous research has often focused on the specific associations of physical exercise on elderly individuals’ cognitive functions, happiness, and physical health, while overlooking the overall impact on SWB (Kumar et al., 2022).

By examining happiness, life satisfaction, and depression as three SWB indicators, this study systematically analyzes multiple pathways through which physical exercise is associated with SWB. This indicator-based analytical approach deepens our understanding of the role of physical exercise and fills a gap in existing research, providing empirical evidence relevant to health intervention strategies in aging societies.

#### 5.1.2 Mediating role of health capital

The empirical analysis of this study reveals that health capital plays a significant mediating role in the relationship between physical exercise participation and SWB among older adults. Specifically, physical exercise is associated with higher health capital among older adults, which is in turn associated with higher happiness and life satisfaction and lower depressive symptoms. This finding is consistent with existing research in the fields of psychology and happiness economics and further expands its theoretical boundaries. A large body of literature suggests that high levels of health capital are often associated with greater happiness and life satisfaction, whereas severe illnesses tend to persistently diminish an individual’s sense of well-being (Ngamaba et al., 2017).

Moreover, older adults with insufficient health capital often face challenges such as social exclusion, stigmatization, and unequal life opportunities, which further erode their life attitudes and mental health (Hansen and Slagsvold, 2016). However, existing research has primarily focused on the direct relationship between health capital and SWB across different indicators, with limited exploration of its mediating role in the relationship between physical exercise and SWB.

This study further demonstrates that older adults who engage in physical exercise show higher levels of health capital. Their higher self-rated health status is associated not only with better physical functioning and disease prevention capacity but also with better mental health, such as lower depressive symptoms, lower psychological stress, and higher life positivity. This finding not only enriches the theoretical framework of health capital in aging research but also provides scientific evidence relevant to constructing a health-oriented elderly care service system.

#### 5.1.3 Mediating role of social capital

This study finds that social capital plays a significant mediating role in the relationship between physical exercise and older adults’ SWB, a mechanism that can be further elucidated from the perspectives of later-life social relationships and emotional well-being. Physical exercise among older adults is often organized in collective and context-specific forms, such as square dancing, walking groups, and Tai Chi classes. These activities expand older adults’ interpersonal networks through repeated interactions, strengthen neighborhood ties and norms of reciprocity, and foster a heightened sense of belonging, trust, and acceptance (Qu et al., 2023). In line with activity theory and social participation theory, meaningful and sustained social participation helps older adults maintain a sense of role continuity and social integration, thereby being associated with higher life satisfaction and overall SWB (Baeriswyl and Oris, 2023).

In later life, as health functions decline and social roles change, the social capital of older adults faces the risk of continuous erosion, with some individuals experiencing a sense of “uselessness” (Zhao et al., 2017). This psychological state not only affects their perception of the aging process but also is associated with weaker emotional experiences, thus threatening their SWB. Previous studies have pointed out that older adults, through active participation in social activities, can gain a sense of recognition and achievement, which is associated with maintaining vitality (Cacioppo and Hawkey, 2009).

Therefore, physical exercise, as an important means for older adults to adapt to society, is associated with higher social capital, and in turn is associated with lower social isolation and lower perceived social exclusion and loneliness, thereby being associated with better SWB. This finding not only enriches the practical application of social capital theory but also provides scientific evidence relevant to developing healthy aging strategies centered around social participation.

#### 5.1.4 Mediating role of psychological capital

This study also provides evidence consistent with psychological capital as another crucial pathway through which physical exercise is associated with the SWB of older adults. Psychological capital encompasses positive psychological states such as self-efficacy, hope, resilience, and optimism, and plays a critical role in helping older adults cope with stress and maintain emotional stability (Jurek and Niewiadomska, 2021), further validating its applicability in the context of aging.

From the perspective of self-efficacy, regular exercise enables older adults to accumulate “successful experiences” in the process of achieving exercise goals, thereby strengthening their belief in their own capabilities (Toros et al., 2023). This enhanced sense of competence may be associated with other life domains, relating to higher perceived control and happiness. From the perspectives of resilience and optimism, physical exercise typically takes place in a “stress–adaptation” context, in which older adults learn to adjust their pace and persevere in the face of physical fatigue and difficulties (Toth et al., 2024). This process may help to build the capacity to “bounce back” from adversity and to maintain positive expectations about the future. Empirical studies have shown that older adults with higher levels of psychological resilience tend to benefit more from physical activity and maintain better cognitive functioning and emotional states (O'Doherty et al., 2024).

In addition, psychological capital may interact with social capital: social support networks can strengthen individuals’ self-efficacy and optimism, while older adults with higher psychological capital are more likely to engage actively in group activities, thereby expanding their social capital. In this study, the two types of capital were examined as parallel mediating pathways, and their potential synergistic mechanisms were not explored in depth, which points to a promising direction for future research.

#### 5.1.5 Heterogeneity in the associations between physical exercise and the SWB of older adults

This study, based on subgroup regressions by gender, urban/rural residence, and marital status, finds that the positive association between physical exercise and the SWB of older adults shows some variation in point estimates across groups. In terms of happiness, the association is relatively larger in magnitude for males, rural residents, and unmarried older adults. This may stem from men’s greater emphasis on “capability affirmation” through exercise—strengthening physical fitness and self-efficacy (Aaltonen et al., 2020). Rural areas generally lag in the overall supply of public resources. Rural seniors often achieve both basic health management and daily social interaction through regular physical exercise, a relatively low-cost approach (Ji et al., 2025). Unmarried older adults, lacking emotional support from a spouse, may rely more on non-family networks (such as exercise communities) to gain a sense of belonging (Zhang J. et al., 2024).

For the life satisfaction indicator, the association is relatively larger in coefficient estimates for females, rural residents, and unmarried elderly individuals. On one hand, females tend to engage in group-oriented, highly interactive sports formats, fulfilling relational needs and emotional expression through frequent social interactions during physical activities, thereby being associated with higher overall life evaluations (Golaszewski et al., 2022). For rural residents, common collective activities not only improve physical health but also rebuild social capital through neighborhood interactions, thereby potentially strengthening the association with SWB. For unmarried older adults or those lacking family companionship, group-based physical activity may provide more regular social contact and a sense of belonging and is linked to reduced loneliness and higher life satisfaction; accordingly, the estimated exercise–life satisfaction association may appear larger in this subgroup (Wu et al., 2024).

For the depression indicator, the results show that female, urban, and unmarried older adults exhibit relatively larger negative coefficient estimates between physical exercise and depression scores. Compared with men, older women generally have higher baseline levels of depressive symptoms and are more prone to emotional distress, so they may show a larger exercise–depression index association through emotional catharsis and enhanced social support (Guo et al., 2024). Urban older adults are exposed to greater life stress and more prolonged sedentary behavior, making the stress-relieving and antidepressant associations of physical activity more salient in urban contexts (Liu et al., 2023). The decline in depression index scores appears larger among unmarried older adults, suggesting that, in the relative absence of spousal or family support, physical exercise may play a potentially important compensatory role in providing emotional support and social connectedness.

### 5.2 Recommendations

First, given the robust positive association between physical exercise and older adults’ subjective well-being, promoting physical activity can be viewed as a scalable well-being intervention within healthy ageing agendas. Policy emphasis should shift from short-term advocacy to routine provision by improving the accessibility and continuity of exercise opportunities, so that regular activity can be more easily integrated into daily life, particularly in resource-constrained communities and areas.

Second, because health capital plays a key mediating role in the exercise–well-being relationship, physical activity promotion should be more closely integrated with primary care and chronic disease management rather than delivered as an isolated leisure initiative. In practice, strengthening a closed-loop arrangement involving risk assessment, personalized guidance, and ongoing support can align exercise participation with functional maintenance and risk management, thereby consolidating the health foundation through which well-being gains are realised.

Third, as social capital constitutes an important pathway, communities and public spaces should be treated as central settings for building social connectedness, and intervention design should prioritize sustained social participation and the formation of supportive ties. This calls for investment in organizational capacity and sustainable operation on the supply side, ensuring continuity, inclusiveness, and low barriers to participation, so that exercise not only improves physical functioning but also consistently generates interaction and perceived support, potentially amplifying subjective well-being benefits.

Fourth, given the mediating role of psychological capital, exercise promotion can adopt a mind–body orientation by embedding light-touch, standardized, and replicable psychological support elements into routine activity settings to enhance positive affect and psychological resilience. Correspondingly, basic capacity building for front-line organizers and service providers is important so that psychological benefits can be reinforced alongside physical and social improvements.

Fifth, subgroup regressions suggest potential variations in associations at the level of point estimates. Policy design should therefore combine universal provision with an equity-oriented approach through proportionate targeting, directing additional support to older adults with relatively weaker resources or social support, such as those living in underserved areas or lacking family support. Meanwhile, heterogeneity signals should be evaluated continuously and refined adaptively during implementation; monitoring and feedback can guide resource reallocation to balance equity, feasibility, and effectiveness across different institutional and community contexts.

## 6 Research limitations and future prospects

### 6.1 Generalizability across cultural and institutional contexts

The conclusions are based on older adults in China, and the regional cultural background and policy environment may limit generalizability to other countries or welfare systems. Future research could examine the cross-cultural robustness of the proposed mediation patterns through cross-country comparisons (e.g., high-welfare countries versus emerging economies).

### 6.2 Psychometric implications of single-item measurement for the mediators

Although we identify indirect associations through health, social, and psychological capital, these mediators are primarily measured with single-item self-reports in the CFPS, which may shape the boundaries of association estimation and mechanism interpretation. Single-item measures do not allow reliability to be assessed and may attenuate coefficients, making indirect effects more likely to be underestimated; they also capture global evaluations and may not fully represent the multidimensional nature of these constructs, and common-method variance may bias estimates when outcomes are also self-reported. Future work could incorporate validated multi-item scales or richer indicators and apply latent-variable approaches to explicitly address measurement error.

### 6.3 Potential omitted variables and model fit

Potential determinants of older adults’ SWB—such as social support, living environment, and economic shocks—are not fully incorporated, which may lead to omitted-variable bias and relatively low model fit (R^2^). It should be noted that SWB measures typically involve substantial individual heterogeneity and self-reported measurement noise, and are also influenced by unobserved and relatively stable factors (e.g., personality traits and affective baselines); therefore, modest R^2^ values are common in the SWB literature. Accordingly, a low R^2^ is better interpreted as reflecting the multifactorial nature of SWB outcomes and the presence of unobserved components, rather than as a standalone basis for dismissing the direction or statistical significance of the estimated associations. Future research may expand key covariates while also leveraging more exogenous shocks or stronger identification strategies to further assess robustness and improve model fit.

### 6.4 Boundaries of heterogeneity assessment and multiple-comparison concerns

Subgroup regressions are used to describe the direction and magnitude of the association between physical exercise and subjective well-being across populations. Differences in subgroup coefficients mainly reflect point-estimate variation; due to multiple testing, extensive difference testing may increase the risk of false positives. Future research may incorporate pooled interaction models with Wald/bootstrapped tests and multiple-testing adjustments to provide more rigorous evidence on between-group differences.

### 6.5 Study design and directionality

Given the study design and the predominance of self-reported measures, the three survey waves are analyzed as pooled cross-sections rather than using individual fixed effects or cross-lagged panel models. Accordingly, the ability to account for time-invariant individual characteristics and to assess directionality remains constrained. Future studies with longer follow-up and more fine-grained measures could employ fixed-effects or dynamic panel approaches.

## Data Availability

Publicly available datasets were analyzed in this study. This data can be found at: the datasets generated and analyzed during the current study were derived from the China Family Panel Study (CFPS). They are opened to everyone. Researchers who want to use these data can visit www.isss.pku.edu.cn/cfps/.
